# Interactions Between Gastroesophageal Reflux Disease and Diabetes Mellitus: A Systematic Review of Pathophysiological Insights and Clinical Management Strategies

**DOI:** 10.7759/cureus.66525

**Published:** 2024-08-09

**Authors:** Kishor Kumar, FNU Bhawana, FNU Vandna, FNU Pirya, Pirya Kumari, Anjlee Sawlani, Sara Sara, FNU Simran, Ankash Kumar, FNU Deepa, Ali Gul

**Affiliations:** 1 Internal Medicine, Liaquat University of Medical and Health Sciences, Karachi, PAK; 2 Internal Medicine, Peoples University of Medical and Health Sciences for Women, Nawabshah, PAK; 3 Internal Medicine, Liaquat National Hospital and Medical College, Karachi, PAK; 4 Internal Medicine, Dow University of Health Sciences, Karachi, PAK; 5 Internal Medicine, Northern Lincolnshire and Goole NHS Foundation Trust, Goole, GBR; 6 Internal Medicine, Chandka Medical College, Larkana, PAK; 7 Internal Medicine, Ghulam Muhammad Mahar Medical College, Sukkur, PAK; 8 General Surgery, Nishtar Medical University, Multan, PAK

**Keywords:** therapeutic strategies, autonomic neuropathy, delayed gastric emptying, clinical management, pathophysiological mechanisms, diabetes mellitus, gastroesophageal reflux disease

## Abstract

This systematic review elucidates the complex interplay between gastroesophageal reflux disease (GERD) and diabetes mellitus, integrating findings from various studies to highlight pathophysiological connections and effective clinical management strategies. Our examination reveals that mechanisms such as delayed gastric emptying and autonomic neuropathy significantly contribute to the exacerbation of GERD symptoms in diabetic patients, influencing clinical outcomes and treatment efficacy. The review underscores the necessity of multidisciplinary approaches in treating these comorbid conditions and advocates for therapeutic strategies that simultaneously address GERD and diabetes, such as the use of prokinetic agents and tailored surgical interventions like laparoscopic Roux-en-Y gastric bypass. This synthesis advances our understanding and proposes a foundation for future research and clinical practice, aiming to improve the quality of life and treatment outcomes for affected patients. This work contributes significantly to gastroenterology and endocrinology, providing a comprehensive resource for clinicians and researchers alike.

## Introduction and background

Gastroesophageal reflux disease (GERD) and diabetes mellitus are two prevalent chronic conditions that pose significant health burdens globally. GERD, characterized by the reflux of stomach contents into the esophagus, affects approximately 20% of Western populations, with similar rising trends observed worldwide [1]. Diabetes mellitus, a metabolic disorder marked by chronic hyperglycemia, is associated with a myriad of complications affecting nearly every system in the body, including the gastrointestinal tract [2]. The interplay between diabetes and GERD is particularly complex, as diabetes can exacerbate or contribute to the development of GERD through mechanisms such as gastroparesis, which delays gastric emptying, and autonomic neuropathy, which impairs gastrointestinal motility [3].

The clinical management of patients suffering from both conditions is challenging, as the conventional therapeutic strategies for one may aggravate the other. For example, some prokinetic drugs used in GERD management may affect glucose control, complicating diabetes management [4]. Moreover, the standard surgical interventions for severe GERD and obesity-related issues in diabetic patients, such as gastric bypass or sleeve gastrectomy, carry risks and benefits that must be carefully weighed [5]. Understanding the pathophysiological mechanisms underlying the association between GERD and diabetes is crucial for developing targeted therapies that can manage both conditions effectively without adverse interactions [6].

This systematic review delves into the significance of the co-occurrence of GERD and diabetes, exploring how the exacerbation of one can influence the progression and treatment of the other. By investigating the pathophysiological pathways, such as the impact of hyperglycemia on lower esophageal sphincter function and the role of autonomic dysfunction in gastroparesis, this review addresses critical gaps in the current literature that has previously outlined associations without deep exploration of causative mechanisms [3,4]. Our primary objective is to synthesize existing research on the interactions between GERD and diabetes mellitus, focusing on the pathophysiological mechanisms and clinical management strategies. This review aims to delineate the complex relationship between these two diseases and to evaluate the efficacy and safety of various treatment modalities. By integrating findings from diverse studies, we seek to offer novel insights into how these conditions intersect and propose more effective, holistic management strategies for patients suffering from GERD and diabetes mellitus. This review not only aims to enhance clinicians' understanding but also to guide future research directions in this intersecting field of gastroenterology and endocrinology, urging further investigation into integrated treatment approaches and the long-term outcomes of these complex interrelations.

## Review

Materials and methods

Search Strategy

Our search strategy was rigorously developed in accordance with the Preferred Reporting Items for Systematic Reviews and Meta-Analyses (PRISMA) guidelines to thoroughly investigate the interplay between GERD and diabetes mellitus, emphasizing pathophysiological mechanisms and clinical management strategies. We conducted comprehensive searches across major electronic databases including PubMed, Medline, Embase, the Cochrane Library, and Google Scholar, covering literature from the inception of each database up to March 2024 to incorporate the latest research findings.

We utilized a precise combination of keywords and Medical Subject Headings (MeSH) terms aligned with our research objectives, such as "gastroesophageal reflux disease," "GERD," "diabetes mellitus," "pathophysiology," "clinical management," and "treatment outcomes." Boolean operators ("AND", "OR") were applied to refine and enhance the specificity of our search queries. Example search strings included: "GERD AND diabetes mellitus AND clinical outcomes," "gastroesophageal reflux disease AND type 2 diabetes AND treatment strategies," and "esophageal dysfunction OR gastric motility AND diabetic patients." To ensure comprehensive coverage, we also reviewed reference lists of selected articles and included clinical trial registries and relevant conference proceedings to identify unpublished or ongoing studies. Our search was limited to publications in peer-reviewed journals, focusing on clinical trials, cohort studies, case-control studies, and systematic reviews that elucidate the interactions between GERD and diabetes mellitus, particularly their pathophysiological connections and management strategies.

Eligibility Criteria

The eligibility criteria for this systematic review are meticulously defined to ensure the inclusion of precise and pertinent studies on the intersection of GERD and diabetes mellitus. We focus on peer-reviewed research articles, including clinical trials, cohort studies, case-control studies, and systematic reviews, that examine the pathophysiological mechanisms and clinical management strategies for these conditions.

Inclusion criteria are strictly set to encompass only peer-reviewed studies exploring pathophysiological links or evaluating clinical management strategies for GERD and diabetes mellitus. This includes pharmacological treatments, lifestyle modifications, and surgical interventions, covering studies published from the databases' inception until March 2024.

Exclusion criteria aim to enhance the review's relevance by omitting studies that do not specifically address the co-management or pathophysiological overlap of GERD and diabetes mellitus. We exclude animal studies and grey literature (including conference abstracts and unpublished theses) to maintain scientific rigor and reliability. Additionally, studies lacking detailed descriptions of relevant interventions or outcomes are not considered, ensuring clarity and depth in our systematic review.

Data Extraction

Our data extraction process was meticulously designed to ensure the accuracy and completeness of data for our systematic review of the interactions between GERD and diabetes mellitus, focusing on pathophysiological insights and clinical management strategies. Initially, two independent reviewers screened article titles and abstracts. Each article was categorized based on its relevance to our research objectives: "relevant" articles directly addressed the intersection of GERD and diabetes with clear pathophysiological or management insights; "not relevant" articles did not pertain to the specific interaction between these conditions; and "potentially relevant" articles contained indirect or tangential information that might influence the review's scope upon deeper investigation. Articles deemed potentially relevant underwent comprehensive full-text review using a standardized data extraction form in Microsoft Excel (Microsoft® Corp., Redmond, WA, USA), capturing essential information such as author, publication year, study design, sample size, outcomes, findings, and limitations. Discrepancies between reviewers were resolved through discussion or arbitration by a third reviewer, ensuring consistency and objectivity. This systematic approach facilitated thorough analysis and synthesis of relevant data, enabling robust conclusions about GERD and diabetes mellitus in clinical settings.

Data Analysis and Synthesis

Given the diverse methodologies and outcomes of the studies reviewed, a meta-analysis was deemed unsuitable for examining the interactions between GERD and diabetes mellitus. Instead, we employed a structured qualitative approach to more effectively dissect and understand the pathophysiological mechanisms and clinical management strategies. Our analytical process involved a detailed thematic analysis where key findings, such as delayed gastric emptying and autonomic dysfunction, were categorized into distinct themes. This approach enabled us to systematically identify and discuss the underlying patterns and trends across studies, emphasizing the impacts of various pharmacological and surgical treatments.

To ensure rigor and transparency, each step of our narrative synthesis followed a predefined protocol. This protocol outlined the procedures for data extraction, theme identification, and synthesis, ensuring that all data were treated consistently. We also employed cross-validation techniques among reviewers to verify theme categorizations and to ensure that the synthesis captured all relevant aspects of the interaction between GERD and diabetes. This comprehensive overview not only highlighted the current research landscape but also discussed the implications of integrated treatment strategies. We pinpointed significant literature gaps, particularly concerning long-term treatment outcomes and comparisons of different management strategies. These insights not only contribute to more effective patient management but also set the stage for future research that can explore these identified gaps in greater depth.

Results

Study Selection Process

**Figure 1:**
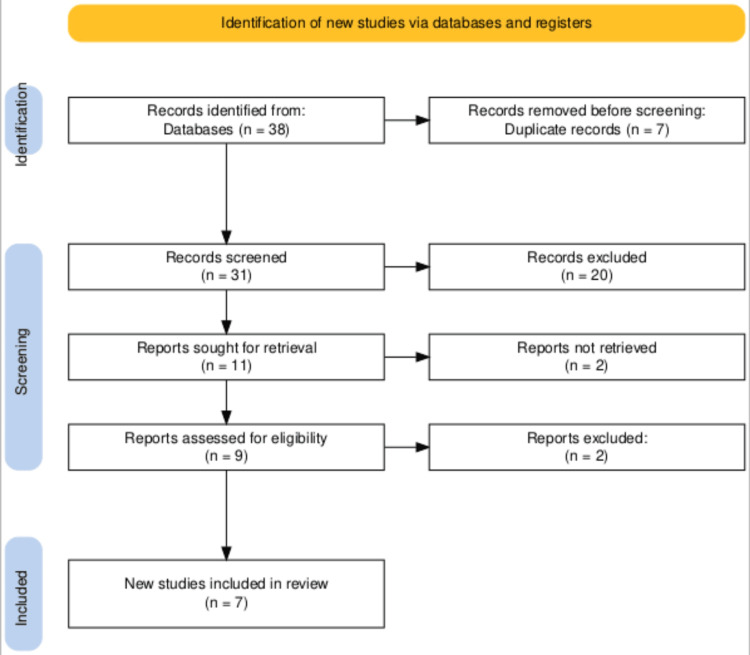
PRISMA flow diagram illustrating the study selection process for the systematic review. PRISMA: Preferred Reporting Items for Systematic Reviews and Meta-Analysis

The search across various databases and registers initially yielded 38 records. After deduplication, which removed seven duplicate entries, 31 records were screened for relevance. This screening process led to the exclusion of 20 records due to irrelevance to the study criteria, leaving 11 reports that were sought for detailed evaluation. Of these, two reports could not be retrieved, and the remaining nine were thoroughly assessed for eligibility. After this assessment, two reports were further excluded for failing to meet the inclusion criteria, resulting in seven new studies being included in the systematic review. The PRISMA flowchart provided in Figure 1 visualizes this study selection process clearly and methodically, ensuring transparency and reproducibility of the study inclusion process.

Characteristics of Selected Studies

Our systematic review included seven studies illuminating the complex relationship between GERD and diabetes mellitus. Salminen et al.’s multicenter randomized controlled trial (RCT) [6] in Finland compared laparoscopic sleeve gastrectomy (LSG) and laparoscopic Roux-en-Y gastric bypass (LRYGB) surgeries in 240 patients, highlighting better outcomes with LRYGB. Emile et al.’s review [7] of single-anastomosis sleeve ileal (SASI) bypass in 941 patients noted significant weight loss and low complication rates. Quast et al.’s study [8] on 57 diabetics found that lixisenatide effectively delays gastric emptying, improving the management of gastric functions. Woodman et al.’s [9] study on adjustable gastric banding (AGB) in 171 obese patients with GERD reported significant improvements in symptoms and quality of life. Fedorchenko et al.’s trial [10] among 90 diabetics showed itopride superior to domperidone in treating GERD. Koch et al.’s registry analysis [11] on 137 diabetic patients assessed gastroparesis severity, identifying different outcomes between diabetes types. Finally, Kinekawa et al.’s study [12] on eight diabetics using aldose reductase inhibitor (ARI) epalrestat demonstrated improved esophageal function. These studies collectively enhance the understanding of treatment impacts and suggest a tailored approach to managing both conditions effectively. These details are given in Table 1.

**Table 1:** Summary of the key studies included in the systematic review. LSG: laparoscopic sleeve gastrectomy; LRYGB: laparoscopic Roux-en-Y gastric bypass; RCT: randomized controlled trial; %EWL: percent excess weight loss; SASI: single-anastomosis sleeve ileal; BMI: body mass index; GLP-1 RAs: glucagon-like peptide-1 receptor agonists; AGB: adjustable gastric banding; GERD: gastroesophageal reflux disease; APEX: adjustable gastric banding postmarket approval clinical study; IP: itopride; DP: domperidone; T1DM: type 1 diabetes mellitus; T2DM: type 2 diabetes mellitus; GI: gastrointestinal; ARI: aldose reductase inhibitor

Lead Author	Objective	Design	Setting	Participants	Interventions	Main Outcomes	Results	Conclusions
Salminen et al. (2022) [6]	Compare long-term outcomes of LSG and LRYGB	10-year multicenter equivalence RCT	Finland, 2008-2021	240 patients, aged 18-60	LSG vs. LRYGB	%EWL, comorbidities remission, esophagitis, Barrett's esophagus	LRYGB had a greater %EWL; LSG had more esophagitis	Both surgeries provided sustainable weight loss, LRYGB had better outcomes
Emile et al. (2021) [7]	Review outcomes of SASI bypass	Systematic review	Literature review	941 patients from 10 studies	SASI bypass	%EWL, BMI change, comorbidities, complications	SASI resulted in significant weight loss and comorbidity improvement	SASI bypass effective with low complication rate
Quast et al. (2020) [8]	Effects of GLP-1 RAs on esophageal reflux and gastric function	Randomized controlled trial	10-week clinical trial	57 type 2 diabetic patients	Lixisenatide vs. Liraglutide	Reflux episodes, esophageal sphincter pressure, gastric emptying	No significant effect on reflux episodes; lixisenatide delayed gastric emptying	Lixisenatide better at delaying gastric emptying
Woodman et al. (2010) [9]	Effect of AGB on GERD and quality of life	Prospective longitudinal study	Prospective APEX study	171 obese patients with GERD	Adjustable gastric banding (AGB)	GERD symptoms, quality of life, comorbidities improvement	Significant improvements in GERD and quality of life	AGB significantly improved GERD and quality of life
Fedorchenko et al. (2013) [10]	Effectiveness of itopride vs domperidone in GERD treatment in diabetics	Comparative, randomized controlled trial	Clinical trials with strict randomization	90 diabetic patients with GERD	Itopride (IP) vs domperidone (DP)	GERD symptom relief, endoscopic findings, gastric motility	IP is more effective than DP in treating GERD	Itopride recommended over Domperidone for treating GERD in diabetics
Koch et al. (2016) [11]	Compare gastroparesis severity in T1DM vs T2DM	Comparative analysis within a registry	Multicenter study at seven centers	137 diabetic patients with gastroparesis	Observational assessment	Gastroparesis severity, healthcare utilization, quality of life	T2DM showed improvement in GI symptoms, not T1DM	Different outcomes in T1DM vs T2DM suggest other mediating factors
Kinekawa et al. (2005) [12]	Effect of ARI on esophageal dysfunction in diabetics	Interventional study	Monitoring esophageal pH and motility	Eight type 2 diabetic patients	ARI epalrestat (150 mg/day) for 90 days	Esophageal pH and motility	Significant improvement in esophageal function	ARI effective for esophageal dysfunction in diabetic patients

Discussions

Our systematic review delved into the intricate relationship between GERD and diabetes mellitus, synthesizing findings from diverse studies to clarify pathophysiological interactions and refine clinical management strategies. Salminen et al.’s [6] research demonstrated the differential impacts of LSG and LRYGB, showing LRYGB's superiority in terms of weight loss and lower esophagitis rates.

Emile et al. [7] highlighted the efficacy of SASI bypass in achieving significant weight loss with minimal complications, suggesting its suitability for obese diabetic patients. Quast et al.’s [8] trial revealed that lixisenatide, a glucagon-like peptide-1 (GLP-1) receptor agonist, effectively delays gastric emptying, potentially improving GERD management in diabetic patients. Woodman et al.’s [9] study on AGB noted marked improvements in GERD symptoms and quality of life, presenting AGB as a viable treatment option for obese individuals with diabetes. In contrast, Fedorchenko et al. [10] found that itopride more effectively alleviated GERD symptoms compared to domperidone, offering a preferable therapeutic profile.

Koch et al.’s [11] research within a registry noted distinct effects of diabetes type on gastrointestinal symptoms, with type 2 diabetes patients experiencing more symptom improvement than those with type 1. Finally, Kinekawa et al.’s [12] study on the ARI epalrestat significantly enhanced esophageal function in diabetics, providing a valuable approach for managing esophageal dysfunction in this group. These studies collectively advance our understanding of managing GERD in the context of diabetes, advocating for personalized treatment approaches.

Previous literature has established a complex relationship between diabetes and increased risk of GERD, often attributing this to factors such as autonomic neuropathy and delayed gastric emptying, which are prevalent in diabetic individuals [13]. Our findings align with these studies, particularly highlighting the impact of diabetes on gastric motility. However, our review extends these observations by demonstrating how specific interventions, like the use of GLP-1 receptor agonists and surgical options such as SASI bypass and LRYGB, modulate these pathophysiological factors, offering therapeutic benefits beyond typical GERD and diabetes management [14].

The review also addresses gaps in the literature, especially in the nuanced understanding of how different surgical interventions impact GERD outcomes in diabetic patients. While previous research has often shown mixed results regarding the benefits of bariatric surgery on GERD symptoms in diabetic patients, our synthesis clarifies these discrepancies. Salminen et al.’s work [6] provides compelling evidence of the differential outcomes of LSG and LRYGB on GERD, a topic less frequently addressed in depth in earlier studies.

To further contextualize the clinical significance of the interplay between GERD and diabetes mellitus, it is essential to consider their epidemiological profiles. GERD affects approximately 20% of the Western population, with prevalence rates showing a similar upward trend in various global regions, reflecting changes in dietary patterns, obesity rates, and aging populations [15]. On the other hand, diabetes mellitus continues to be a major global health concern, with an estimated 537 million adults living with the condition worldwide as of 2021, a number projected to rise to 643 million by 2030. The convergence of these two prevalent conditions underscores the critical need for integrated clinical approaches, particularly as the overlap between them increases the complexity of management and impacts patient quality of life significantly [16].

The understanding of the relationship between GERD and diabetes has significantly evolved over the past few decades. Initial studies focused primarily on the isolated pathophysiological mechanisms of each condition without considering their potential interactions. Seminal research in the late 20th century began to shed light on the bidirectional influences of diabetes and gastrointestinal function, noting that diabetic autonomic neuropathy could exacerbate GERD symptoms by impairing gastric motility [17]. Subsequent studies have built on these findings, exploring how hyperglycemia directly affects the lower esophageal sphincter's functioning, thus increasing the risk of acid reflux [18].

This evolving understanding has gradually shifted clinical practices from treating GERD and diabetes as entirely separate entities to a more integrated approach. For example, the recognition of delayed gastric emptying in diabetics has led to the more frequent use of prokinetic agents in managing GERD among this population, highlighting the importance of tailored treatment strategies that address both metabolic control and gastrointestinal health [19].

Furthermore, our analysis reveals novel insights into the effectiveness of itopride over domperidone in managing GERD among diabetics, an area that has not been extensively covered. This finding could guide more targeted pharmacological strategies in clinical practice, emphasizing a tailored approach to managing comorbid GERD and diabetes [20].

Our systematic review elucidates several key pathophysiological mechanisms that link GERD and diabetes mellitus, enhancing our understanding of how these conditions may exacerbate each other. Central to this interrelationship is the impact of diabetes on gastric motility - primarily through diabetic gastroparesis - which prolongs gastric emptying times, thereby increasing the risk of gastroesophageal reflux. This mechanism is critical as it not only intensifies typical GERD symptoms but also complicates glucose control, creating a challenging cycle of symptom management. Moreover, autonomic neuropathy, prevalent in diabetes, impairs the motility of the gastrointestinal tract and the function of the lower esophageal sphincter, further predisposing individuals to reflux [21].

The interaction between these mechanisms and clinical outcomes is significant. For instance, treatments that improve gastric emptying can also alleviate reflux symptoms, as evidenced in studies comparing the effects of GLP-1 receptor agonists. Such mechanistic understanding helps explain the varied efficacy of treatments across patients with differing severities of diabetic complications and provides a rationale for personalized treatment approaches [22].

Additionally, the differential effects of various bariatric procedures on GERD symptoms call for careful preoperative assessment and patient counseling regarding the expected outcomes related to both weight loss and reflux symptoms. Surgeons and clinicians should consider these factors when planning intervention strategies, potentially steering patient selection for specific types of surgeries based on their pre-existing conditions and severity of symptoms [23].

Furthermore, the review brings to light new theoretical models for understanding the interaction between diabetes and GERD, emphasizing a multidisciplinary approach to treatment that considers the patient as a whole rather than in isolated conditions. This holistic approach could pave the way for integrated care models where endocrinologists, gastroenterologists, and dietitians work together to devise comprehensive management plans that address metabolic control and gastrointestinal health [24].

While informative, the studies in our review present several limitations that could impact the interpretation of our findings. Many studies had small sample sizes and short follow-up periods, which may not adequately capture the long-term outcomes and effectiveness of treatments for GERD and diabetes, potentially leading to an overestimation of treatment efficacy or underreporting of long-term complications [25]. Additionally, the lack of diversity in patient populations across these studies may limit the generalizability of the findings to broader populations, as outcomes could vary significantly across different ethnic, age, and socio-economic groups [26].

Furthermore, inconsistencies in study designs and methodologies contribute to challenges in comparing data across studies. This variance can obscure the true effectiveness of treatment strategies and impede the development of universally applicable management guidelines. To enhance the reliability and applicability of future research outcomes, it is crucial to include larger, more diverse populations and utilize standardized methods that can provide more robust and comparable data.

Given the gaps identified in our review, future research should focus on long-term, longitudinal studies that evaluate the effectiveness of integrated treatment approaches for managing GERD and diabetes simultaneously. There is a particular need for RCTs that compare different pharmacological and surgical treatments to establish clearer guidelines for clinical practice [27]. Research questions could explore how modifications in lifestyle and dietary interventions could complement pharmacological or surgical treatments to improve outcomes in these patients. Additionally, exploring the genetic and molecular bases of the interaction between diabetes and GERD could unveil targeted therapies and personalized treatment plans [28].

The findings from our review have significant broader implications beyond the immediate clinical management of GERD and diabetes. Understanding the link between these conditions could inform more effective screening programs and preventive measures for public health, potentially reducing healthcare costs and improving quality of life. These insights could lead to more informed dietary recommendations and lifestyle modifications tailored to patients with both conditions. Moreover, integrating these findings into clinical guidelines can enhance multidisciplinary care approaches, encouraging collaboration among specialists in endocrinology, gastroenterology, and dietetics to offer holistic patient care [29,30].

## Conclusions

This systematic review has delineated the complex interplay between GERD and diabetes mellitus, revealing critical insights into their pathophysiological connections and the effectiveness of various treatment modalities. The findings underscore the necessity for a multidisciplinary approach in the clinical management of patients suffering from both conditions, highlighting the potential benefits of integrated therapeutic strategies that address the unique challenges posed by the coexistence of GERD and diabetes. Future research should continue exploring innovative treatments and refining management protocols, ensuring that clinical practices evolve in line with emerging evidence to optimize outcomes for this growing patient demographic. This review enhances our understanding of these interrelated health issues and points to the broader implications for patient care, urging ongoing dialogue and investigation in the intersecting fields of gastroenterology and endocrinology.
